# Analysis of Morphological Traits, Cannabinoid Profiles, *THCAS* Gene Sequences, and Photosynthesis in Wide and Narrow Leaflet High-Cannabidiol Breeding Populations of Medical Cannabis

**DOI:** 10.3389/fpls.2022.786161

**Published:** 2022-02-24

**Authors:** Jana Murovec, Jan Jurij Eržen, Marko Flajšman, Dominik Vodnik

**Affiliations:** Department of Agronomy, Biotechnical Faculty, University of Ljubljana, Ljubljana, Slovenia

**Keywords:** *Cannabis sativa* L., high CBD medical cannabis, cannabinoids, photosynthesis, respiration, *THCA synthase*, morphometry

## Abstract

*Cannabis sativa* L. is one of the oldest cultivated crops, used in medicine for millennia due to therapeutic characteristics of the phytocannabinoids it contains. Its medicinal properties are highly influenced by the chemotype, that is, the ratio of the two main cannabinoids cannabidiol (CBD) and Δ-9-tetrahydrocannabinol (THC). Based on published data, the chemotype should correlate with plant morphology, genetics, and photosynthetic properties. In this work, we investigated leaf morphology, plant growth characteristics, cannabinoid profiles, *THCAS* gene sequences, and plant photosynthetic traits in two breeding populations of medical cannabis (MX-CBD-11 and MX-CBD-707). The populations differed significantly in morphological traits. The MX-CBD-11 plants were taller, less branched, and their leaves had narrower leaflets than the bushier, wideleaved MX-CBD-707 plants, and there were significant differences between populations in the dry biomass of different plant parts. Based on these morphological differences, MX-CBD-11 was designated as a narrow leaflet drug type or vernacular “Sativa” type, while MX-CBD-707 was classified as wide leaflet drug type or “Indica” type. Chemical characterisation revealed a discrepancy between the expected chemotypes based on plant morphology; although both populations have high CBD, within each Type II (CBD/THC intermediate) and Type III (CBD dominant) plants were detected. The *THCAS* gene sequence analysis clustered the plants based on their chemotypes and showed high similarity to the *THCAS* sequences deposited in NCBI. *In silico* complementary analysis, using published molecular markers for chemotype determination, showed their low discrimination power in our two populations, demonstrating the genotype dependence of the molecular markers. Basic photosynthetic traits derived from light and CO_2_ response curves were similar in the populations. However, measurements of gas exchange under chamber conditions revealed higher stomatal conductivity and photosynthesis in MX-CBD-707 plants, which were also characterised by higher day respiration. The results of this study showed that based on visual appearance and some morphological measurements, it is not possible to determine a plant’s chemotype. Visually homogenous plants had different cannabinoid profiles and, vice versa, morphologically distinct plants contained similar CBD and THC content. The two chemotypes identified in our experimental plants therefore did not correlate with plant visual appearance, leaf morphometry, and photosynthetic properties of the populations studied. Correlation was only demonstrated with the respect to *THCAS* sequences, which showed great discrimination power between the chemotypes.

## Introduction

Cannabis (*Cannabis sativa* L.) is gaining popularity in the modern world through industrial, food, cosmetic, and medicinal uses. It is one of the oldest cultivated crops, having been grown worldwide for a plethora of purposes for millennia. This has led to the development of numerous groups of plants that, although genetically and phenotypically diverse, can interbreed and are therefore difficult to classify based on standard botanical nomenclature. Hence, various types of classifications have been introduced over the past century.

A generally accepted classification of cannabis plants is based on their primary agronomic purpose, which determines the traits to be selected and consequently profoundly affects the phenotypes of registered varieties. The most widely cultivated group is “hemp” (“fibre-type hemp,” “industrial cannabis”), which was once an important crop for the production of raw materials for textiles and ropes and which is currently experiencing a revival after a steady decrease in its acreage after World War II (Tang et al., 2017). It is grown for seeds and fibre, food and beverage production, substances for cosmetic use, animal feed, and other industrial uses. It can be cultivated as a field crop of registered varieties that contain no more than a legally defined, country-specific threshold level of the psychoactive substance Δ-9-tetrahydrocannabinol (THC). In European countries, for example, the threshold is set at 0.2% or 0.3% THC in the upper third of the dried plant or in an upper 30 cm of dried plants shoots containing at least one female inflorescence (Regulation EU No. 809/2014).

Although hemp can also be used for pharmaceutical purposes, it contains small amounts of cannabinoids. Higher relative (in per cent of inflorescence dry weight) and absolute (in g per cultivated m^2^) amounts of cannabinoids can be produced in cannabis varieties popularly known as “medical cannabis” (“marijuana,” “drug type cannabis”). They contain high levels of plant cannabinoids, of which cannabidiol (CBD) and THC are the most abundant and pharmaceutically most important (Friedman et al., 2019). They are produced in secretory cells within glandular trichomes as carboxylic acids cannabidiolic acid (CBD-A) and Δ9-tetrahydrocannabinolic acid (Δ9-THC-A) that are decarboxylated to their corresponding neutral forms CBD and THC, respectively, upon heating (Salami et al., 2020; Tanney et al., 2021). Medical cannabis varieties contain higher amounts of THC than the legal national limits for hemp and can be grown indoors or outdoors only in compliance with strict national legal restrictions.

The relative abundance and ratio of CBD and THC has led to the second most widely used cannabis nomenclature, which divides cannabis plants into three discrete groups: “THC dominant” or “high THC” (CBD/THC ratio 0.00–0.05), “intermediate” (CBD/THC ratio 0.5–3), and “CBD dominant” or “high CBD” (CBD/THC ratio 15–25) (Staginnus et al., 2014). These three chemical phenotypes (chemotypes) have been named Type I (THC dominant), Type II (CBD/THC balanced) and Type III (CBD dominant) (Small and Beckstead, 1973; de Meijer et al., 1992, 2003).

The first systematic genetic analyses of chemotype inheritance, performed by crossing and self-pollination of different chemotypes, indicated simple codominant inheritance through a single locus B with two alleles: the B_*T*_ allele for THCA synthase (THCAS) and the B_*D*_ allele for CBDA synthase (CBDAS). Based on this model, Type II plants would be heterozygous B_*D*_B_*T*_, while the plants of pure chemotypes Type I and III would be homozygous for B_*T*_B_*T*_ and B_*D*_B_*D*_, respectively (de Meijer et al., 2003; Mandolino et al., 2003; Toth et al., 2019). A more complex model of inheritance now prevails. The genetic basis of chemotypes is thought to be determined by at least two closely linked loci, one encoding CBDAS and the other encoding THCAS, in medical and hemp cultivars (de Meijer et al., 2003; Kojoma et al., 2006; van Bakel et al., 2011; Onofri et al., 2015; Weiblen et al., 2015; Grassa et al., 2021) and/or by variation in gene copy number (Weiblen et al., 2015; Vergara et al., 2019). The cannabinoid profile is thought to be determined by the presence of *THCAS* and *CBDAS* with normal, weak, or no expression, resulting in Type I plants containing only functional *THCAS* gene, Type II plants containing functional genes for both synthases, and Type III plants lacking functional copies of *THCAS* and containing functional *CBDAS* (van Bakel et al., 2011; Weiblen et al., 2015). However, as shown by Zirpel et al. (2018a,b), both the *THCAS* and *CBDAS* genes are capable of producing THCA, CBCA and CBDA, as well as five other unknown products, which may explain the occurrence of low THCA levels in Type III cultivars (such as the hemp cultivar “Finola”) carrying only one functional *CBDAS* allele (van Bakel et al., 2011).

The third level of differentiation between cannabis plants is based on their morphology, which is the oldest marker. It was used for plant classification by the pioneers in this field. As comprehensively reviewed by Jin et al. (2021b), Linneaeus described *C. sativa* L. in 1753 in *Species Plantarum* as a plant with loose inflorescences covered with sparse trichomes and resembling a northern European fibre-type landrace. Later, in 1785, de Lamarck described a second (or sub-) species, *Cannabis indica* Lam. collected in India, with dense trichomes, narrower leaflets, a branching habitus, poorer fibre quality, a harder stem, and a thinner cortex, but stronger psychoactive effects. Schultes travelled to Afghanistan in 1971 and described *C. indica* as having wide leaflets, densely branched with very dense inflorescences for hashish (resin) production, departing from Lamarck’s original taxonomic concept. Anderson drew illustrations of *C. indica* and *C. sativa* in 1980. The former was depicted as short, conical, densely branched, and with wide leaflets; the latter as relatively tall, laxly branched, and with narrow leaflets, which agreed with Schultes but diverged from Lamarck. Later, Hilling performed extensive analyses on 157 accessions of different geographic origins, classifying them into two species, *C. sativa* and *C. indica*, and seven putative taxa, including the narrow leaflet drug (NLD) biotype of *C. indica*, the wide leaflet drug (WLD) biotype of *C. indica*, the hemp biotype of *C. indica*, the feral *C. indica* biotype, the hemp biotype of *C. sativa*, the feral *C. sativa* biotype, and putative ruderal populations. The NLD biotype included landraces of Indian heritage (including cultivars from the Indian subcontinent, Africa, and other drug-producing regions) corresponding to Lamarck’s *C. indica*. The WLD biotype included landraces from Afghanistan and Pakistan corresponding to Schultes’*C. indica*. The *C. indica* hemp biotype included landraces from South and East Asia, while the *C. sativa* hemp biotype included landraces from Europe, Asia Minor, and Central Asia.

Because of its complexity, the above classification has not been adopted for everyday use in the cannabis industry and recreational cultivation; therefore, the vernacular expressions “Sativa” and “Indica” have become accepted to describe cultivars with narrow leaflets and broad or wide leaflets, respectively. They were based on illustrations by Anderson, which differed from the original botanical nomenclature. “Sativa” plants produce much more THC than CBD, while “Indica” plants produce almost equal amounts of THC and CBD, with a CBD/THC ratio of around 1 (McPartland, 2017). However, as McPartland also reported, these vernacular categories are unreliable for distinguishing between different chemotypes and/or cannabis end uses due to extensive cross-breeding and incomplete labelling during hybridisation (McPartland, 2017). In addition, in most classification studies, samples had come from different sources and had been exposed to inconsistent environmental factors during growth phases, postharvest treatment, sample preparation, and extraction procedures during laboratory analysis (Jin et al., 2020, 2021a,2021b). Jin et al. (2021b) recently addressed these drawbacks. They analysed phenotypic variation in 21 cannabis cultivars covering three chemical phenotypes (THC dominant, intermediate, and CBD dominant) by measuring 30 morphological traits at the vegetative, flowering, and harvest stages on live plants and harvested inflorescences. Significant morphological differences were found between plants and chemotypes. Among others, leaflets characteristics were found to be usable as phenotypic markers to distinguish THC dominant, intermediate, and CBD dominant cultivars included in their study. Canonical correlation analysis assigned the experimental plants to the corresponding chemotypes with 92.9% accuracy (Jin et al., 2021b).

The physiological distinction of cannabis morphotypes/chemotypes is not clear. In an early study by Bazzaz et al. (1975), differences in the photosynthetic rate and THC content were found in four populations of *C. sativa* from temperate and warm climatic regions. Drug-type and fibre-type cannabis ecotypes tested by Lydon et al. (1987) had similar photosynthetic properties. Chandra et al. (2011) reported considerable variation in the temperature response of photosynthesis in different drug and fibre types of cannabis. However, the variations were more varietal specific compared with the types (drug and fibre). Overall, the photosynthetic response of cannabis types and varieties mainly reflects their inherited prevalence to specific growing conditions, that is adaptation to the particular environment at the sites of origin.

The relationship between photosynthesis and cannabinoid profile/content is not clear-cut. Photosynthesis interferes with secondary metabolism and some researchers have found that the accumulation of secondary metabolites is directly related to the rate of photosynthesis (Mosaleeyanon et al., 2005). However, this relationship was not demonstrated in cannabis when photosynthesis was assessed by measurements of gas exchange. Khajuria et al. (2020) reported a strong negative correlation between THC content and photochemical efficiency and a weak zeaxanthin-dependent component of non-photochemical quenching (NPQ). The authors even suggested that measuring chlorophyll *a* fluorescence could be used as a rapid tool for high-throughput screening of cannabis for its cannabinoid content, as cannabis plants with a higher CBD than THC content offer better protection of the photosynthetic machinery.

Cannabidiol (CBD) has been shown to have therapeutic effects on humans and animals and no psychoactive effects; it even abolishes the psychoactivity and some adverse effects of THC, such as anxiety, tachycardia, and sedation (Romero et al., 2020). As a result, there has been a dramatic increase in CBD-containing supplements in the food and cosmetic industries in recent years, and even greater potential for its pharmaceutical use has been reported (Glivar et al., 2020; Salami et al., 2020). This has encouraged breeding programmes aimed at developing new varieties of medical cannabis with increased and stable CBD content, as well as basic research into the inheritance of specific chemical profiles.

Two breeding populations (MX-CBD-11 and MX-CBD-707) of medical cannabis were included in this study, both showing high CBD yield in industrial production and contrasting phenotypes based on visual examination. The breeding population MX-CBD-11 resembles a narrow leaflet phenotype, while MX-CBD-707 has a wide leaflet phenotype based on the descriptions of Schultes and Anderson. Our first aim was to analyse precisely the morphological characteristics and cannabinoid content of these populations at the individual plant level. This comprehensive characterisation of the gene pool within our breeding programme enabled us to examine the intra- and inter-population variability of our plants and to verify the correlation between morphotype and chemotype. Because the results showed a uniform morphology within the populations alongside contrasting cannabinoid contents (chemotypes), we further analysed the genetic basis of the observed chemical differences by sequencing their *THCAS* genes. In addition, we measured the photosynthetic characteristics of the breeding populations and analysed them with respect to morphological, chemotype and, genetic differences.

## Materials and Methods

The reported research was conducted on two breeding populations (MX-CBD-11 and MX-CBD-707) of medical cannabis (*C. sativa* L.) owned by MGC Pharma Ltd. (United Kingdom). They were studied as part of the project ‘Breeding medical cannabis (*Cannabis sativa* L.)’, which is a collaboration between the Biotechnical Faculty of the University of Ljubljana and MGC Pharma Ltd. (United Kingdom). Plants were grown in a growth room under controlled temperature, humidity, and illumination at the Agronomy Department of the Biotechnical Faculty, University of Ljubljana, Slovenia. The medical cannabis plants were grown in accordance with a research licence granted by the Ministry of Health of the Republic of Slovenia.

Our breeding programme started with two groups of cannabis plants, which differed in morphological appearance. Plants of each group were crossed within the group in separate rooms and at different times to avoid crossing plants from different groups. The obtained progenies were selected for morphological and growth uniformity within each population and for high CBD content at the industrial production level. Only genetically female plants were cultivated, which were crossed and propagated with feminised seeds obtained by manipulating sex expression, as reported in our recent publication (Flajšman et al., 2021). This approach enabled us to develop two feminised high CBD breeding populations of medical cannabis, one corresponding to the “narrow leaflet drug type” (named MX-CBD-11) and the other one to the “wide leaflet drug type” (named MX-CBD-707) phenotype based on plant morphology according to McPartland (2017, 2018).

Ninety-five plants of the MX-CBD-11 and MX-CBD-707 breeding populations were analysed for genetic (di)similarity with microsatellite markers, as reported in our previous study (Mestinšek Mubi et al., 2020). Twelve genetically distinct plants were randomly selected from each population and included in this study. Each of them was obtained from a germinated feminised seed, therefore representing a unique genotype labelled with a code: 11/02, 11/03, 11/05, 11/06, 11/08, 11/13, 11/20, 11/23, 11/24, 11/25, 11/35, 11/40, 707/03, 707/04, 707/06, 707/08, 707/12, 707/14, 707/31, 707/33, 707/36, 707/39, 707/41, and 707/47. A total of 24 plants were grown in 7 L pots filled with fertilised peat substrate Brown 540 W (Kekkilä, Finland). The culturing conditions in the growth chamber were maintained at: 26°C, 55–60% relative humidity (RH), a photoperiod of 18 h light/6 h dark, and a photosynthetic photon flux density (PPFD) of 350–400 μmol m^–2^ s^–1^ (at canopy level) by using 600-W high-pressure sodium (HPS) lamps (Phantom HPS 600W; Hydrofarm, Petaluma, CA, United States). At the vegetative stage, plants were fertilised with a mixture of vegetative fertilizer (NPK 4-1-2) and CalMag (N-Ca-Mg 2-5-2.5) + microelements in a 1:1 proportion. After 15 weeks of vegetative growth, the photoperiod was changed to 12 h light/12 h dark and fertilisation to flowering fertiliser (NPK 1-3-5) and CalMag (N-Ca-Mg 2-5-2.5) + microelements in a 1:1 proportion in order to induce flowering. Plants from the different populations were randomly distributed in the growth chamber.

### Phenotypic Characterisation of the Breeding Populations

Ten weeks after the beginning of the flowering phase, the plants were harvested. The whole aboveground part of the plants (in our experiment consisting of stems, leaves and inflorescences) was separated from the root system and weighted (“Shoot FW”). After drying the plant material to constant weight, the roots were weighted (“Root DW”) and the dried shoots separated into stems and leaves with inflorescences. They were weighted and the sum of “Stem DW” and “Leaf + inflorescences DW” represented the dry mass of the shoot (“Shoot DW”), while the “Plant DW” was calculated as the sum of “Root DW” and “Shoot DW”. These analyses were performed on five plants per breeding population (*N* = 5).

**FIGURE 1:**
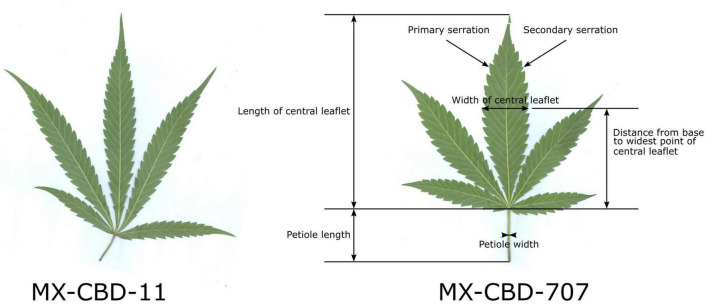
Characteristic leaves of breeding populations MX-CBD-11 (left) and MX-CBD-707 (right) with marked leaf and central leaflet traits that were measured for all the experimental plants included in this study.

The herbarised leaves of all 12 plants per population (*N* = 12) were scanned and several leaf parameters were measured by using CellSense software (Olympus): the number of leaflets per leaf, the length of the central leaflet, the width of the central leaflet, the distance from the base of the central leaflet to the widest point of the leaflet, the petiole length, the petiole width, the number of primary serrations on the central leaflet, and the number of secondary serrations on the central leaflet. Figure 1 shows leaf traits measured on the leaves and their central leaflets.

### Analysis of Cannabinoid Content

The inflorescences were collected 10 weeks after the induction of flowering. Their stems and supporting leaves were removed, and the remaining inflorescences were dried at 40°C to a constant weight. High-performance liquid chromatography (HPLC) analysis was performed in the laboratory of MGC Pharma (Ljubljana, Slovenia) according to Gul et al. (2015) with minor modifications described by Laznik et al. (2020) as follows. Inflorescences were ground at 15,000 rpm for 11 s; then, the cannabinoids were extracted from the plant material by mixing 1 g of powder in 50 mL of methanol (JT Baker) with 0.1% formic acid (Sigma Aldrich) for 30 min at room temperature. The extracts were filtered through 0.45 μm filters (Chromafil^®^ AO -45/25, Macherey-Nagel) and three dilutions were prepared for HPLC analyses. Extracts were analysed by using an Agilent 1260 Infinity quaternary HPLC system with a Poroshell 120 SB -C18 (4.6 × 150 mm, 2.7 μm; Agilent) column. The injection volume was 10 μL and the flow rate was 1,200 mL min^–1^. The oven temperature was 28.0°C and the detection signal wavelength (λ) was 276.0 nm. Mobile phase A was H_2_O (HPLC grade, JT Baker) with 0.1% v/v formic acid, and mobile phase B was acetonitrile (HPLC grade, JT Baker) with 0.1% v/v formic acid. The cannabinoid content of each plant was determined by measuring seven cannabinoids: cannabidiolic acid (CBDA), cannabidiol (CBD), cannabigerolic acid (CBGA), cannabigerol (CBG), Δ8-tetrahydrocannabinol (d8-THC), Δ9-tetrahydrocannabinol (d9-THC), and tetrahydrocannabinolic acid (THCA), using standards specific for each cannabinoid (Cerilliant). A calibration curve was constructed with concentrations ranging from 2 to 75 mg L^–1^ ± 0.006 mg ml^–1^. The concentrations of total CBD, THC, and CBG in % (w/w) were calculated as the sum of the carboxylic acid forms (CBDA, THCA, and CBGA) with the non-carboxylic acid derivatives (CBD, d8-THC, d9-THC, and CBG) using the conversion factor of 0.877 for CBDA and THCA and the conversion factor of 0.878 for CBGA.

### Sequencing the *THCA Synthase* Gene

Genomic DNA was extracted from leaves of each of the 24 mother plants (genotypes) included in this study by using a modified cetyltrimethylammonium bromide (CTAB) method. The full-length *THCA synthase* gene was amplified with the primers THCAsynF (GGA CTG AAG AAA AAT GAA TTG CTC AG) and THCAsynR (GGG AAA TAT ATC TAT TTA AAG ATA ATT AAT GAT) published by Weiblen et al. (2015). Polymerase chain reaction (PCR) was performed in a total volume of 15 μL and contained 25 ng of isolated DNA, 1 × KAPA2G buffer A, 0.8 mM dNTP, 0.5 mM of forward and reverse primers, and 0.3 U Taq DNA polymerase (KAPA2G Fast PCR Kit). The temperature profile was: initial denaturation at 95°C for 3 min; 35 cycles at 95°C for 15 s, 55°C for 15 s, and 72°C for 30 s; and a final extension at 72°C for 10 min. Amplified products were verified on a 1.2% agarose gel electrophoresis and then purified using the GenElute PCR Clean-Up Kit (Sigma-Aldrich Co. LLC). The purified products were cloned into the pGEM®-T Easy Vector (Promega), and the plasmids containing full-length *THCAS* sequences Sanger sequenced using BigDye™ Terminator v3.1 Cycle Sequencing Kit (Applied Biosystems™) according to the manufacturer’s instructions. The sequences were analysed by using CodonCode Aligner (CodonCode Corporation), CLC Genomics (Quiagen) and BLAST algorithm at NCBI. The sequences were first aligned in CLC with default settings and then a genetic distance tree was constructed with Clustal W implemented in CLC.

The obtained *THCAS* sequences were further aligned with primer sequences for published chemotype molecular markers with CodonCode Aligner and CLC Genomics. The aim of this analysis was to determine whether the published molecular markers are suitable to discriminate between Type II and III plants in our breeding populations. The results were scored as complementary or not. To distinguish between different chemotypes, the primers have to be complementary to the *THCAS* gene of only one chemotype of a population. If primers were to anneal to both or none of the chemotypes, such a chemotype marker would be considered non-informative for our breeding populations.

### Photosynthetic Measurements

Gas exchange measurements were performed on the middle leaflet of the first fully developed leaf from the apex (6th or 7th leaf) during the week 15 of the experiment by using the Li-6400xt measuring system (LiCor, Lincoln, NE, United States). Net photosynthesis (*A*), transpiration (*E*), stomatal conductance (*g*_*s*_), leaf intercellular CO_2_ concentration (*C*_*i*_), and photochemical efficiency (F_v_′/F_m_′) were measured by setting Li6400xt controls to growth chamber conditions [PPFD = 400 μmol m^–2^s^–1^, water pressure deficit for leaf (VPDL) 0.9–1.2. kPa; T = 26°C], with reference CO_2_ maintained at 400 μmol mol^–1^. The measurements were done on five plants per breeding population (*N* = 5).

Light response curves of photosynthesis (*AQ* curves; Ögren and Evans, 1993) were measured. The PPFD was varied, keeping the temperature (26°C) and CO_2_ concentration (1000 μmol mol^–1^) constant and controlling the VPDL (1–1.2 kPa). Initially, the measured leaf was acclimated at 1000 μmol m^–2^s^–1^, and after recording the photosynthetic parameters, the light was reduced to 800 μmol m^–2^s^–1^ and later gradually to 600, 400, 200, 100, 50, 0 μmol m^–2^s^–1^. A fast transition of PPFD was used to avoid stomatal closure (Li6400xt manual). Five plants of each line were measured.

The photosynthetic response to CO_2_ (photosynthetic *AC*_*i*_ curves; Sharkey et al., 2007) was evaluated by measuring photosynthetic rates at fixed saturating PPFD (1000 μmol m^–2^s^–1^), a temperature of 26°C, and VPDL of 1–1.2 kPa. The CO_2_ reference concentration was set at 50, 100, 200, 400, 800, 1200, 1400, and 1600 μmol mol^–1^. At each concentration, it took 15–20 min to reach steady-state conditions. Five plants of each line were measured.

Chlorophyll was measured by using the SPAD -502 m (Minolta, Japan) on the leaves sampled for photosynthetic measurements. Six measurements per leaf blade were taken and then averaged. Subsequently, the leaves were sampled and herbarised for morphometry.

### Statistical Analysis

For the basic photosynthetic parameters and those related to leaf morphology and growth, the *t*-test and two-way analysis of variance (ANOVA) were used. We tested the significance of the breeding population only (*t*-test) or in combination with the chemotype and their interaction (two-way ANOVA). *AQ*_*i*_ curves were fitted by using a polynomial quadratic equation (Ögren and Evans, 1993). Photosynthetic light saturation and the light compensation point can be derived from the obtained non-rectangular hyperbolic curve. CO_2_ response curves were fitted and analysed as described by Sharkey et al. (2007), estimating V_cmax_, J, TPU, R_d_ and g_m_ -the maximum rate of carboxylation of Rubisco, the maximum rate of electron transport for the given light intensity, the maximum rate of triose phosphate use, day respiration, and mesophyll conductance for CO_2_ transfer, respectively). For these parameters, differences between breeding populations were tested by using the *t*-test.

A significance level of 0.05 was used for all tests. Data were analysed by using the R environment (packages nlme and agricola; R Core Team, 2018).

## Results

### Morphological Characterization of MX-CBD-11 and MX-CBD-707

The tested breeding populations differed significantly in growth habitus. The MX-CBD-11 plants were taller, less branched, had longer internodes, and had leaves consisting of an average of 5.00 ± 0.21 narrow leaflets. The average length of the central leaflet was 111.59 ± 4.47 mm and the average width was 20.06 ± 0.93 mm.

The MX-CBD-707 plants were shorter, bushier and had shorter internodes. Their leaves had on average 5.08 ± 0.34 leaflets. The average length of the central leaflet was 127.64 ± 6.44 mm, which was not significantly different (*p* = 0.053) from that of breeding population MX-CBD-11. There was a significant difference (*p* ≤ 0.001) for the average width of the central leaflet, which was 26.59 ± 0.71 mm and therefore wider in MX-CBD-707 than in MX-CBD-11 (Figure 1 and Table 1). As a result, the populations differed significantly in the length/width ratio (*p* = 0.038) and the width/length ratio (*p* = 0.020). Besides, the distance from the base of the central leaflet to the widest point of the leaflet was significantly longer in MX-CBD-707 than in MX-CBD-11 (*p* = 0.032) and the petiole width was also significantly wider in MX-CBD-707 than in MX-CBD-11 (*p* = 0.039). Leaf traits were measured for all 24 plants included in our study on fully expanded leaves and their central leaflets, as shown in Figure 1.

**TABLE 1:** Growth and morphological parameters of two breeding populations of medical cannabis, namely MX-CBD-11 and MX-CBD-707.

		MX-CBD-11	MX-CBD-707	p
Plant growth parameters (*N* = 5)	Plant DW [g]	93.2 ± 6.7	59.7 ± 11.2	**0.039**
	Shoot FW [g]	297.6 ± 14.4	198.8 ± 40.1	0.069
	Shoot DW [g]	89.5 ± 6.4	57.5 ± 10.7	**0.039**
	Stem DW [g]	31.9 ± 1.4	14.9 ± 3.4	**0.004**
	Leaf + inflorescence DW [g]	57.6 ± 5.1	42.5 ± 7.6	0.145
	Root DW [g]	3.8 ± 0.5	2.3 ± 0.5	**0.072**
	Shoot/root DW ratio	25.1 ± 3.3	26.8 ± 1.8	0.673

Leaf morphological parameters (*N* = 12)	Number of leaflets per leaf	5.00 ± 0.21	5.08 ± 0.34	0.836
	Length of central leaflet [mm]	111.59 ± 4.47	127.64 ± 6.44	0.053
	Width of central leaflet [mm]	20.06 ± 0.93	26.59 ± 0.71	** < 0.001**
	Length/width ratio of central leaflets	5.65 ± 0.25	4.83 ± 0.27	**0.038**
	Width/length ratio of central leaflets	0.18 ± 0.01	0.21 ± 0.01	**0.020**
	Distance from base to widest point of central leaflet [mm]	56.47 ± 2.71	65.08 ± 2.60	**0.032**
	Distance from base of central leaflet to widest point/total length ratio	0.50 ± 0.01	0.51 ± 0.01	0.521
	Number of primary serrations on central leaflet	28.67 ± 1.11	26.50 ± 1.07	0.174
	Number of secondary serrations on central leaflet	3.08 ± 1.25	2.08 ± 0.54	0.471
	Petiole length [mm]	23.60 ± 2.73	29.47 ± 2.44	0.123
	Petiole width [mm]	0.88 ± 0.05	1.06 ± 0.07	**0.039**

*The data are presented as the mean ± standard error (N = 5 or 12). The p values of the t-tests are shown, with statistically significant p values in bold. DW, dry weight; FW, fresh weight.*

Differences in plants habitus were reflected in yield parameters. For all biomass parameters [shoot dry weight (DW), DW of leaves and inflorescences, stem DW, and root DW], the values were higher in MX-CBD-11 than in MX-CBD-707 (Table 1). Our results of leaf morphology and growth confirmed the assumption that MX-CBD-11 resembles the “narrow leaflet” type of cannabis, while MX-CBD-707 resembles the “wide leaflet” type of cannabis.

### Cannabinoid Profiles of MX-CBD-11 and MX-CBD-707 Breeding Populations

**FIGURE 2:**
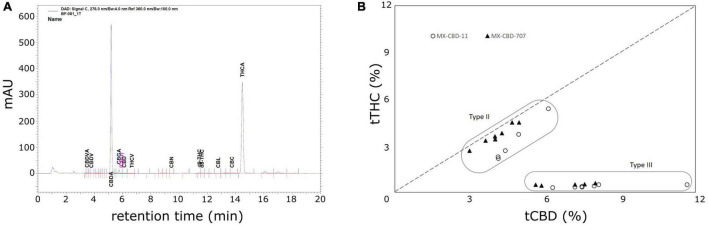
The results of high-performance liquid chromatography (HPLC) analysis of dried inflorescences of breeding populations MX-CBD-11 and MX-CBD-707: **(A)** A representative HPLC chromatogram showing the retention times of various cannabinoids from MX-CBD-707/plant 41; **(B)** total cannabidiol (tCBD) and total tetrahydrocannabinol (tTHC) contents (in % w/w) in all 24 analysed cannabis plants. Each dot represents the measurements of one inflorescence per plant.

High-performance liquid chromatography (HPLC) analysis (Figure 2A) revealed that the cannabinoid content in inflorescences of breeding population MX-CBD-11 varied from 4.11 to 11.66% for tCBD (total CBD) and from 0.31 to 5.39% for tTHC (total THC), while in breeding population MX-CBD-707, the tCBD content varied from 2.99 to 8.01% and the tTHC content varied from 0.42 to 4.49%, as shown in Figure 2B and Table 2.

**TABLE 2:** Concentrations of CBDA, CBD, tCBD, THCA, d8-THC, d9-THC, and tTHC (in % w/w) measured in dried inflorescences of breeding populations MX-CBD-11 and MX-CBD-707.

Breeding population	Cannabinoid	Min [%]	Max [%]	Average retention time [s] ± standard error
MX-CBD-11	CBDA	4.521	12.910	5.185 ± 0.002
(*N* = 12)	CBD	0.125	0.344	6.097 ± 0.002
	tCBD	4.114	11.657	/
	d9-THC	0.029	0.273	11.424 ± 0.002
	d8-THC	0.033	0.175	11.596 ± 0.002
	THCA	0.308	5,631	14.467 ± 0.002
	tTHC	0.310	5.386	/

MX-CBD-707	CBDA	3.293	8.468	5.193 ± 0.002
(*N* = 12)	CBD	0.102	0.582	6.108 ± 0.003
	tCBD	2.990	8.008	/
	d9-THC	0.065	0.363	11.443 ± 0.003
	d8-THC	0.040	0.150	11.606 ± 0.003
	THCA	0.358	4.726	14.487 ± 0.003
	tTHC	0.424	4.492	/

*CBDA, Cannabidiolic Acid; CBD, Cannabidiol; tCBD, total CBD; THCA, Tetrahydrocannabinolic acid; d8-THC, Delta-8-Tetrahydrocannabinol; d9-THC, Delta-9-Tetrahydrocannabinol; tTHC, total THC; / – information non-relevant.*

Within each breeding population, two types of plants were identified based on the tCBD/tTHC ratio: plants with a ratio around 1 (average values 1.52 ± 0.09 in MX-CBD-11 and 1.12 ± 0.01 in MX-CBD-707) and plants with a ratio > 11 (average values 20.09 ± 0.70 in MX-CBD-11 and 14.41 ± 0.44 in MX-CBD-707). The two defined groups of each breeding population were characterised as Type II (CBD/THC balanced) and Type III (CBD dominant) cannabis, respectively.

The tCBG content ranged from 0.05 to 0.27% and from 0.03 to 0.78% in MX-CBD-11 and MX-CBD-707, respectively.

Based on the results obtained from cannabinoid content measurements, a two-way ANOVA of leaf morphological parameters was carried out, considering the breeding population and chemotype as factors. The analysis showed that both the breeding population (*p* < 0.001) and the chemotype (*p* = 0.017) had a significant effect only on the central leaflet width (26.59 ± 0.71 mm for MX-CBD-707 and 20.06 ± 0.93 mm for MX-CBD-11; 25.23 ± 1.00 mm for the CBD/THC balanced chemotype and 21.42 ± 1.29 mm for the CBD-dominant chemotype). For all the other measured parameters listed in Table 1, the chemotype did not have a significant influence. Therefore, a *t*-test was used to analyse the measured parameters.

The tTHC content measured in experimental plants of MX-CBD-11 and MX-CBD-707 demonstrated that they can all be characterised as drug-type cannabis, as none of them contained less than 0.3% tTHC in inflorescence dry weight.

### Sequence Analysis of the *THCAS* Gene in MX-CBD-11 and MX-CBD-707 Breeding Populations

Amplification with the primer pair THCAsynF and THCAsynR resulted in a single approximately 1,676 base pair (bp) PCR product from plants of breeding populations MX-CBD-11 and MX-CBD-707 (Supplementary Figure 1). PCR products were cloned in the pGEM-T-Easy Vector and isolated plasmids were sequenced with the primer pair SP6 and T7, which annealed to the vector backbone. Backbone sequences were removed in CodonCode Aligner and the remaining *THCAS* sequences was aligned in CLC Genomics with standard settings. Alignment of the sequences obtained from MX-CBD-11 revealed several single nucleotide substitutions among the sequences of different plants and clearly grouped the 12 analysed plants into two distinct groups: five Type II plants (CBD/THC balanced) in one group and the remaining seven Type III plants (CBD dominant) in the second group. A consensus sequence from each group was extracted by using CLC and compared with BLASTN to the sequences deposited in NCBI. The consensus sequence of the Type II plants of MX-CBD-11 breeding population showed similarities with several *THCAS* sequences deposited in NCBI and 100% identity with the complete coding DNA sequences (cds) of accessions AB057805, MW382908 and the partial cds of accessions AB212832, KT875984, and MG996418. The consensus sequence of the III chemotype plants was 100% identical and showed 98% overlap with the *tetrahydrocannabinolic acid synthase (THCA2)* gene of the Skunk #1 cultivar (KJ469379, complete cds) and more than 99% identity with several other deposited THCAS sequences [KJ469380 (high CBD cultivar Carmen), MG996405 (high CBD cultivar Ermes1), AB212830, etc.].

We obtained similar results by aligning *THCAS* sequences from MX-CBD-707. The consensus sequences of the Type II and III plants were also compared with the sequences deposited in NCBI. The Type II plants showed 100% identity with five *THCAS* (AB057805, MW382908, AB212832, KT875984, and MG996418), while the Type III plants showed almost complete identity with *THCAS* accessions MT338560, MW504064 (high-THC cultivar Animal Cookies), MW504063 (high-THC cultivar Cake Breath), KT876015, KT875987, and MG996417.

**FIGURE 3:**
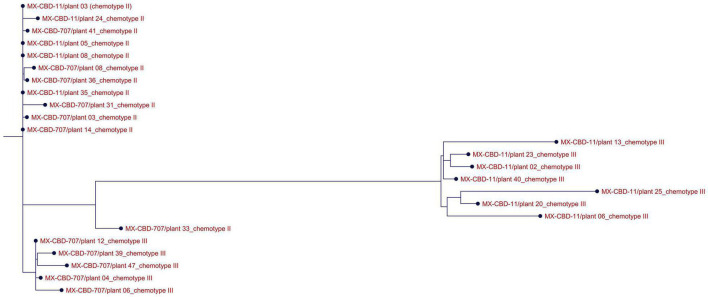
Alignment of *THCAS* sequences from 24 cannabis plants of the MX-CBD-11 and MX-CBD-707 breeding populations with ClustalW. The provenances of the sequences are indicated with the name of the breeding population, the plant’s unique code, and the plant’s chemotype.

ClustalW analysis of all 24 *THCAS* sequences grouped the Type III MX-CBD-11 and MX-CBD-707 plants in two separate clusters, while almost all sequences of Type II plants of both breeding populations were grouped in the same cluster (Figure 3). The only exception was plant 707/33.

To determine whether a PCR and electrophoresis analysis with published molecular markers could be used to distinguish between Type II and III MX-CBD-11 and MX-CBD-707 plants, we performed an *in silico* complementary analysis. It was based on sequence alignment of primers for published chemotype molecular markers to our *THCAS* sequences with CodonCode Aligner and CLC Genomics (Supplementary Figure 1). The results are shown in Table 3 in which “Yes” indicates that the primers are complementary to *THCAS* sequences of the concerned group of plants (e.g., primers D589 to MX-CBD-11 Type II), while “No” indicates that the primers are not complementary due to INDELs or substitutions in the annealing region of the gene. To distinguish between different chemotypes, the primers have to be complementary (should anneal to *THCAS* during PCR) to the DNA of only one chemotype of the population. Primers that are complementary to the DNA of both chemotypes of a population, would amplify a fragment of *THCAS* in both and therefore would not be informative. On the other hand, a primer pair that is not complementary to the DNA of any chemotype is also not informative for the distinction between chemotypes. Such results are marked with an asterisk in Table 3. The chemotypes of MX-CBD-11 could be distinguished by using markers D589 and B1080/B1192, but not THCA583-For/THCA1034-Rev, as these were complementary to the *THCAS* genes of both chemotypes. In contrast, only the primer pair THCA583-For/THCA1034-Rev could be used to distinguish Type II or III MX-CBD-707 plants, whereas the other two (D589, and B1080/B1192) were complementary to all *THCAS* sequences and were therefore not suitable for discrimination between plants of different chemotypes. Primers for marker B190/B200 were not complementary to any of our *THCAS* sequences.

**TABLE 3:** Published DNA molecular markers developed for determination of cannabis chemotypes and their applicability to discriminate between different chemotypes of MX-CBD-11 and MX-CBD-707.

Marker	Reference	Primer sequence	MX-CBD-11		MX-CBD-707	
			Type II	Type III	Type II	Type III
D589	Staginnus et al. (2014)	For CCTGAATTCGACAATACAAAATCTTAGATTCAT	Yes	No	Yes	Yes*
		Rev ACTGAATATAGTAGACTTTGATGGGACAGCAACC	Yes	No	Yes	Yes*
B1080/B1192	Pacifico et al. (2006)	For AAGAAAGTTGGCTTGCAG	Yes	No	Yes	Yes*
		THCAS-specific-Rev TTAGGACTCGCATGATTAGTTTTTC	Yes	No	Yes	Yes*
B190/B200	de Meijer et al. (2003)	For TGCTCTGCCCAAAGTATCAA	No*	No*	No*	No*
		Rev CCACTCACCACTCCACCTTT	No*	No*	No*	No*
THCA583-For	Weiblen et al. (2015)	For GTG GAG GAG GCT ATG GAG C	Yes	Yes*	Yes	Yes*
THCA1034-Rev		Rev CCC AAC TCA GGA AAG CTC TTG	Yes	Yes*	Yes	No

Asterisks (*) mark discrepancies in the expected versus obtained results, because primers D589, B1080/B1192, and THCA583-For/THCA1034-Rev should amplify parts of the functional THCAS gene, while marker B190/B200 should amplify parts of the THCAS (190 bp) and CBDAS (200 bp) genes.

### Photosynthetic Parameters

**FIGURE 4:**
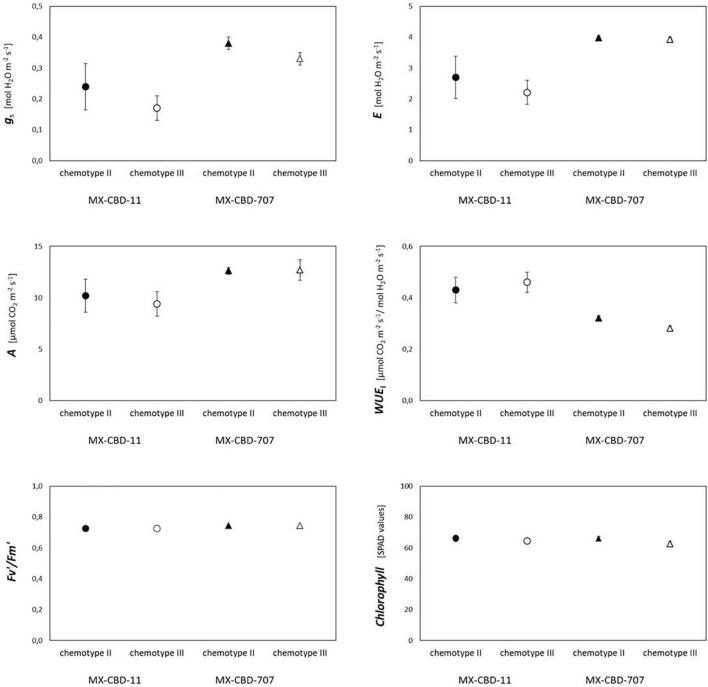
Photosynthetic traits of two breeding populations of medical cannabis MX-CBD-11 and MX-CBD-707. The data are presented as the mean ± standard error (*N* = 5).

Most photosynthetic parameters differed significantly between breeding populations, while they were not dependent on chemotype (Table 4). Stomatal conductance (*g*_*s*_), transpiration (*E*), and net photosynthesis (*A*) measured under chamber conditions were higher in MX-CBD-707 than in MX-CBD-11 (Figure 4). However, intrinsic water use efficiency (*WUE*), calculated as the ratio of *A* to *E*, was higher in MX-CBD-11 than in MX-CBD-707. There was no difference in chlorophyll content (SPAD).

**TABLE 4:** The results of two-way analysis of variance (ANOVA) (factors: breeding population, chemotype) for stomatal conductance (*g*_*s*_), transpiration (*E*), net photosynthesis (*A*), intrinsic water use efficiency (*WUE*_*i*_ = *A*/*E*), photochemical efficiency, and chlorophyll content (SPAD) (*N* = 5).

ANOVA (p-value)	*g*_*s*_	*E*	*A*	*WUE*	*F*_v_′/*F*_m_′	Chlorophyll (SPAD)
Breeding population	**0.004**	**0.008**	**0.025**	**0.001**	0.052	0.467
Chemotype	0.210	0.446	0.716	0.971	0.999	0.105
Breeding population × chemotype	0.821	0.633	0.715	0.357	0.941	0.523

*Statistically significant p values are presented in bold.*

**FIGURE 5:**
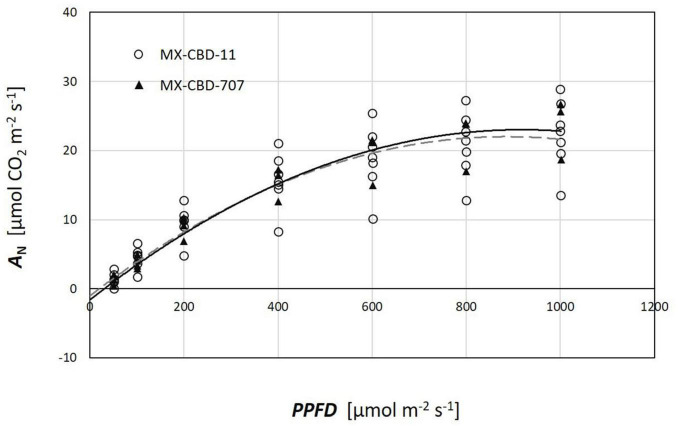
Photosynthetic light response (*AQ*) curves of two breeding populations of medical cannabis: MX-CBD-11 and MX-CBD-707. The data points indicate measurements on individual plants (5 plants of each population).

Plants from both populations did not differ in photosynthesis dependence on light (Figure 5). The *AQ* curves showed a similar photosynthetic light compensation point, similar light use efficiency (the slope of the initial linear part of the curve), and similar light saturation. Photosynthesis of both populations was light saturated above 600 μmol m^–2^s^–1^.

**FIGURE 6:**
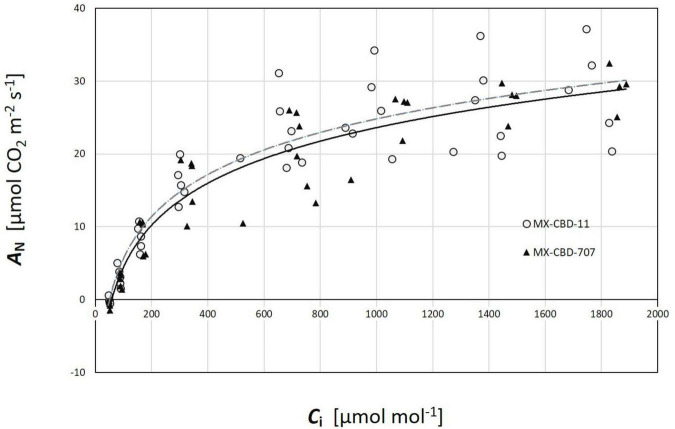
Photosynthetic CO_2_ response (*AC*_*i*_) curves of two breeding populations of medical cannabis MX-CBD-11 and MX-CBD-707. The data points indicate measurements on individual plants (5 plants of each population).

Regarding CO_2_ response curves, the maximum photosynthetic rates were measured at 2000 μmol CO_2_ mol^–1^ (under saturating PPFD of 1000 μmol m^–2^s^–1^), and were 37.1 and 36.7 μmol m^–2^s^–1^ for MX-CBD-11 and MX-CBD-707, respectively. Analysis of the *AC*_*i*_ curves (Figure 6) showed that plants from the two populations did not differ in the maximum carboxylation rate of Rubisco (V_*cmax*_), the maximum rate of electron transport (J), and the maximum rate of triose phosphate utilisation (TPU) (Table 5). However, differences in day respiration (R_*d*_) were pronounced, with MX-CBD-707 (R_*d S*707_ = 3.9 ± 0.6 μmol m^–2^s^–1^) having significantly higher respiration than that of the MX-CBD-11 breeding population (R_*d S*11_ = 1.9 ± 0.5 μmol m^–2^s^–1^).

**TABLE 5:** Maximum carboxylation rate of Rubisco (V_*cmax*_), maximum rate of electron transport (J), maximum rate of triose phosphate utilization (TPU), and day respiration (R_*d*_) of two breeding populations of medical cannabis MX-CBD-11 and MX-CBD-707.

	V_cmax_	J	TPU	R_d_
MX-CBD-11	100.2 ± 24.4	122.3 ± 12.0	9.9 ± 0.9	1.9 ± 0.5
MX-CBD-707	133.5 ± 15.7	137.2 ± 11.4	11.0 ± 0.9	3.9 ± 0.6
*t*-test	ns	ns	ns	*p* = 0.0338

*The data are presented as the mean ± standard error (N = 5).*

**FIGURE 7:**
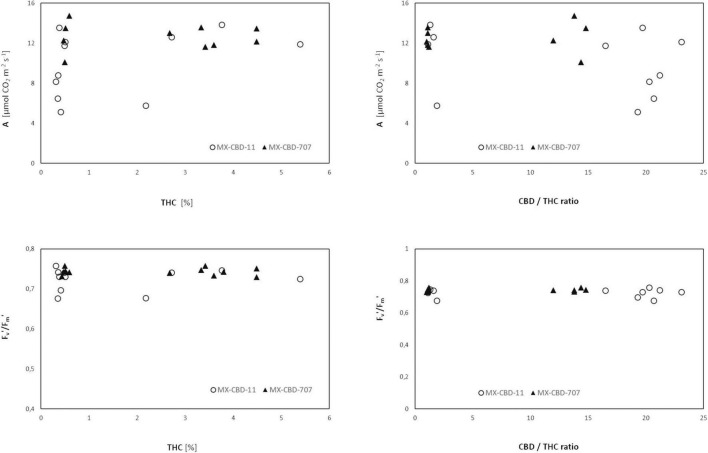
Net photosynthesis (*A*) and photochemical efficiency *F*_v_′/*F*_m_′ of two breeding populations of medical cannabis MX-CBD-11 and MX-CBD-707 as a function of cannabinoid content.

Comparison of cannabinoid content (expressed as the THC content or the CBD/THC ratio) and photosynthesis [net photosynthetic rate (*A*), assessed by gas exchange] or photochemical efficiency (*F*_v_′/*F*_m_′, assessed by fluorescence measurements) revealed no clear relationship (Figure 7).

## Discussion

The chemical profiles of CBD-dominant (Type III) and intermediate (Type II) cannabis chemotypes are gaining increased attention due to the therapeutic potential without psychoactive effects of CBD (Avraham et al., 2011). As a result, numerous breeding programmes are underway aimed at increasing CBD content and stabilising this trait in breeding populations intended for varietal registration. Plant genetic resources are searched for accessions suitable for introgression of this valuable trait in breeding programmes, and plant phenotypes are often used as morphological markers.

It has long been assumed that cannabis plants can be divided into a few groups/ecotypes whose specific phenotypes correlate with the plant’s chemotypes. Since the early work of Linnaeus in 1753, several contrasting classifications of cannabis have been proposed. Fossil pollen studies suggest that genetic drift initiated allopatric differences between European *C. sativa* and Asian *C. indica*. *C. sativa* and *C. indica* could thus be separated by morphology (*C. sativa* is taller with a fibrous stalk, whereas *C. indica* is shorter with a woody stalk) and, by phytochemistry (*C. sativa* with THC > CBD, whereas *C. indica* with THC < CBD). DNA barcode analysis supports the separation of these taxa at a subspecies and not species level, recognising the formal nomenclature of *C. sativa* subsp. *sativa* and *C. sativa* subsp. *indica* (McPartland, 2018). In the same publication, a diverse description of the (sub)species is listed (subsp. *sativa* containing < 0.3% of THC and subsp. *indica* containing > 0.3% THC in dried inflorescences). When considering other authors, the classification/nomenclature and descriptions of cannabis are even more confusing. As pointed out by McPartland (2017), the ubiquitous interbreeding and hybridisation of cannabis species, subspecies, and ecotypes in recent decades renders their distinction almost meaningless. As a result, vernacular taxonomy of drug-type plants “Sativa” and “Indica” prevails today: cannabis plants are classified primarily on the basis of leaf morphology. The narrow leaflet drug-type plants (the “Sativas”) can be identified by their narrow and light green leaves and should produce more THC than CBD, while deep green and wide leaflet drug-type plants (“Indicas”) should produce more CBD than “Sativa,” with a THC/CBD ratio closer to 1. “Indica” refers to plants with broad leaflets, compact habitus, and early maturation, typified by plants from Afghanistan. “Sativa” refers to plants with narrow leaflets, slender and tall habitus, and late maturation, typified by plants from India and their descendants in Thailand, South and East Africa, Colombia, and Mexico (Figure 4 in McPartland, 2018). The author emphasised that conflating formal and vernacular taxonomy has resulted in the confusion of otherwise excellent studies that used “Sativa” but latinised the taxon as *C. sativa*.

We therefore decided to study the morphology, chemotype, genotype, and physiology of two cannabis breeding populations that based on visual appearance showed characteristics of NLD and WLD plants. Furthermore, we wanted to determinate the linkage between morphological and chemical traits and to use the obtained data to classify our cannabis populations based on literature data. As recently reported in an excellent study in which Jin et al. (2021b) phenotypically characterised 21 cannabis cultivars covering three chemical phenotypes, morphological traits can be used reliably to distinguish among cannabis chemotypes, which facilitates taxonomic classification.

We first measured plant growth and leaf parameters, which confirmed uniformity within populations and showed significant differences between populations. Among the measured parameters, plant, shoot, stem, and root dry weights showed statistically significant differences between MX-CBD-11 and MX-CBD-707, with clear differences in biomass distribution and a higher biomass accumulation in MX-CBD-11. Interestingly, there were no significant differences in dry weights of inflorescences and leaves.

A detailed analysis of leaf morphology showed statistically significant differences in the average width of central leaflets (*p* < 0.001), the distance from the base of the central leaflet to the widest point of leaflets (*p* = 0.032), and the petiole width (*p* = 0.039) between the two studied populations. These differences were reflected in the calculated ratios of central leaflet width to length and distance from the base to the widest point divided by the total length, which were further compared with the results reported by Jin et al. (2021b). The calculated mean value of the central leaflet width/length ratio of MX-CBD-11 was 0.18 ± 0.01, which is identical to the value measured by Jin et al. (2021b) for CBD-dominant cultivars (0.18 ± 0.02), while MX-CBD-707 had a wider average central leaflet width with a higher width/length ratio of 0.21 ± 0.01. The calculated ratio was between their parameters for CBD dominant (0.18 ± 0.02) and THC dominant (0.25 ± 0.03) cultivars, most similar to the ratio of intermediate plants (0.20 ± 0.02). Moreover, Jin et al. (2021b) demonstrated that the CBD-dominant cultivars have more leaflets per leaf (4.45–5.39, average 4.92 ± 0.47) compared with the intermediate and THC-dominant cultivars. In our study, both breeding populations had a similar average number of leaflets per leaf, namely 5.0 for MX-CBD-11 and 5.1 for MX-CBD-707, both resembling CBD-dominant cultivars. Our results confirmed the ones reported by Jin et al. (2021b), because our breeding populations were considered high CBD at an industrial production scale.

The calculated ratio of distance from the base of the central leaflet to the widest point divided by the total length was 0.50 ± 0.01 and 0.51 ± 0.01 for MX-CBD-11 and MX-CBD-707, respectively, and did not differ significantly (*p* = 0.521). Jin et al. (2021b) obtained nearly identical results: 0.50 and 0.51 for all three chemotype groups of cultivars, without a significant difference among them (*p* = 0.9282). Absolute values of measured leaf parameters were less comparable between our study and Jin et al. (2021b) and therefore less applicable for chemotype determination.

Because there was a discrepancy in some leaf (leaflet) traits, we could not fully rely on morphological classification by Jin et al. (2021b) to deduce the cannabinoid profile of the plants. Moreover, on the basis of plant habitus, we would classify MX-CBD-11 as “Sativa” because the plants were taller, had longer internodes, and had light green narrow leaflets, while MX-CBD-707 would be classified as “Indica” because the plants were shorter, bushier, and had deep green wide leaflets. Based on vernacular classifications, MX-CBD-11 should contain higher THC than CBD (“Sativa”) and MX-CBD-707 more CBD than MX-CBD-11, with a THC/CBD ratio closer to 1.

We proceeded with the analysis of cannabinoids to verify their content in the narrow leaflet MX-CBD-11 and the wide leaflet MX-CBD-707 breeding populations. We sampled and processed inflorescences from each experimental plant separately to obtain results at the individual plant level rather than as population averages presented in other publications and our previous analyses of these two populations. HPLC measurements of the main cannabinoids revealed that plants within both populations differed significantly in their cannabinoid content. Within the MX-CBD-11, the minimum and maximum values of total CBD and total THC varied by 2.84- and 18.35-fold, respectively. A 3.87- and 16.26-fold difference in tCBD and tTHC was observed in MX-CBD-707 plants (Figure 2B). Calculating the tCBD/tTHC ratio allowed us to identify plants of two different chemotypes within each population. The tCBD/tTHC ratios varied from 1.04 to 23.14 and classified the plants of both breeding populations into Type II (CBD/THC intermediate) with an average ratio of 1.52 ± 0.09 (MX-CBD-11), and 1.12 ± 0.01 (MX-CBD-707), and Type III (CBD dominant), with an average ratio of 20.09 ± 0.70 (MX-CBD-11) and 14.41 ± 0.44 (MX-CBD-707) (Figure 2B). In contrast to the reports by Welling et al. (2016), higher variability in cannabinoid composition was observed in Type III plants compared with Type II plants in our study. The cannabinoid contents in our breeding populations were unexpected because plants within populations had consistent phenotypes based on visual inspection and leaf measurements. At least for our NLD and WLD populations, the results obtained disprove the theory about the correlation between plant morphology and cannabinoid content. This was further analyzed with a two-way analysis of variance in which we tested the influence of the breeding population, the chemotype and their interaction on leaf morphology. The analysis showed that the chemotype had a significant effect only on the average width of the central leaflet (*p* = 0.017), while not to the other measured or calculated leaf parameters presented in Table 1. It also confirmed a significant effect of the breeding population to the width of the central leaflet (*p* < 0.001), the length to width ratio of the central leaflets (*p* = 0.044), the width to length ratio of the central leaflets (*p* = 0.021), the distance from the base of the central leaflet to the widest point of the leaflet (*p* = 0.023) and the petiole width (*p* = 0.048), like it was already shown with the *t*-test (Table 1). The chemotype (*p* = 0.292) or the interaction (*p* = 0.502) did not have a significant effect on the ratio of central leaflet width to length, as was also reported by Jin et al. (2021b). In our experiment, the average value was larger in Type II (CBD/THC intermediate) plants than in Type III (CBD dominant) ones (0.21 ± 0.01 and 0.19 ± 0.01, respectively), which was also in accordance with the results of Jin et al. (2021b; 0.20 ± 0.02 for Type II and 0.18 ± 0.02 for Type III). Similarly, like reported in Jin et al. (2021b), our average values of distances from the base to the widest point divided by the total length were not significantly different between the two chemotypes (*p* = 0.056).

Because both populations had similar ranges of tCBD and tTHC and both contained Type II and III plants, we wanted to determine whether the chemotypes from different populations were determined by the same alleles. We sequenced the *THCAS* gene because, according to the literature, both Type II and III plants contain functional alleles for *CBDAS*, so we did not expect to find differences in that gene. Type II plants should also contain a functional *THCAS*, while Type III plants should be caused by non-functionality of *THCAS*. The genes for *THCAS* were amplified from all 24 plants that were included in our study and sequenced using classical Sanger sequencing. Alignment of the obtained sequences correlated with their tCBD/tTHC ratios (Figure 3), with Type III MX-CBD-11 and MX-CBD-707 plants clustering in two distinct groups and all but one Type II plant from both breeding populations clustering together as one group. BLASTN analysis of *THCAS* gene sequences showed high (up to 100%) similarity with *THCAS* sequences deposited in NCBI. Interestingly, we found 100% similarity between the consensus sequence of MX-CBD-11-chemotype III plants and the *THCAS* gene from cultivar Skunk #1 (KJ469379). This was unexpected because previous findings suggest that Type III plants contain non-functional alleles for *THCAS*, while Skunk #1 is a hybrid cultivar with high THC content (Type I). The same consensus sequence showed > 99% identity with several other deposited *THCAS* sequences, two of which were from high-CBD cultivars of both drug and fibre types (KJ469380 drug type Carmen and MG996405 fibre type Ermes1). One of our *THCAS* sequences was outside its group based on chemotype (Figure 3). This ambiguity was due to poorer sequence quality, with gaps and unknown nucleotides caused by sequencing errors.

Molecular markers are also widely used to determine the chemotypes of cannabis. In recent years, several molecular DNA markers based on the analysis of bulk segregants of *THCAS* and *CBDAS* gene sequences have been developed to allow rapid and accurate determination of plant chemotypes in marker-assisted selection. They relied on the model of simple genetic determinism of chemotypes based on a gene with two alleles encoding two isoforms (*THCAS*, and *CBDAS*) of the same enzyme, as described by de Meijer et al. (2003). Two dominant (D589, THCA583-For/THCA1034-Rev) and two codominant (B1080/B1192, B190/B200) markers have been described in the literature (Table 3) and have been used successfully to determine chemical types. For B190/B200, de Meijer et al. (2003) showed 88% correct identification of Type I chemotypes, 95% for Type II, and 98% for Type III, while Pacifico et al. (2006) used the B190/B200 marker to determine the chemotypes of 148 plants and obtained only 20% accuracy for Type I, 0% for Type II, and 93% for Type III. They developed a new codominant marker B1080/B1192 that gave 100% correct identification. Welling et al. (2016) used a combination of two markers (D589 and B1080/B1192) and accurately predicted the chemotype of > 98% of plants (65 of 66). Our *in silico* complementary analysis showed that the published molecular markers were not equally effective in unrelated plant material with different genetic backgrounds. As shown in Table 3, only the THCA583-For/THCA1034-Rev marker could be used to discriminate between Type II and III MX-CBD-707 plants, whereas the other three could amplify parts of the *THCAS* genes in all MX-CBD-707 plants. For MX-CBD-11, markers D589 and B1080/B1192 could be used, but not THCA583-For/THCA1034-Rev and B190/B200. This simple analysis clearly demonstrated genotype dependence of the developed molecular markers.

There was no clear relationship between biomass yield and photosynthesis in either breeding line. High maximum photosynthetic rates indicate that the plants were grown under suitable conditions. The higher photosynthesis (*A*) and transpiration (*E*) measured in MX-CBD -707 plants can be attributed to higher stomatal conductance (*g*_*s*_). As a result, plants in this line operated at a slightly lower water use efficiency compared with MX-CBD-11 plants. In general, the results of gas exchange measurements indicate a different stomatal regulation of the two lines under growth chamber conditions. The values for V_*cmax*_, J, and TPU derived from the and *AC*_*i*_ curves were within the range reported by Tang et al. (2017) for moderately nitrogen-supplied hemp. There were no differences between the breeding populations, even when comparing the light response curves. However, leaf photosynthetic performance under chamber conditions was better in MX-CBD-707 than in MX-CBD-11, which, in contrast, had a higher biomass yield. This discrepancy could be explained by differences in carbon allocation. The different plant habitus and biomass accumulation patterns of the tested populations suggest differences in the distribution of photosynthates to different sinks, plant parts, ephemeral, and long-lived tissues. In addition, a significant fraction of carbohydrates may be used for respiration. The twofold higher day respiration (R_*d*_) of the MX-CBD-707 population could reduce photosynthetic gain of carbohydrates and, consequently, lead to lower biomass accumulation. Significant differences in leaf respiration between different cannabis cultivars (fibre and drug type) were previously reported by Lydon et al. (1987). More detailed analyses would be required for a deeper understanding of allocation, including analyses of mechanical tissue (fibre content) and non-structural carbohydrates.

Neither chlorophyll content (i.e., greenness) nor photochemical efficiency, which have been reported as possible indicators of cannabinoid profile (Khajuria et al., 2020; Jin et al., 2021b), were associated with the CBD/THC ratio. This calls into question the use of physiological parameters for chemical screening of cannabis.

## Conclusion

The species *C. sativa* L. exhibits an astonishing diversity of morphological, physiological, and chemical characteristics, all of which could be attributed to the species great genetic diversity and adaptation to different growing conditions. Previously published scientific data have shown correlations between chemotype categories and traits of the plant phenotype, genes encoding cannabinoid synthesising enzymes, and physiology. However, our study has shown that the reported correlations are genotype dependent and apply to the genotypes included in the reported studies. The two chemotypes identified in our experimental plants did not differ in plant visual appearance, leaf morphology, and photosynthetic traits in the populations studied. Correlation was only demonstrated with the respective *THCAS* sequences, which showed great discrimination power between the chemotypes, whereas previously published molecular markers for chemotype determination were not found to be equally reliable in a different genetic background.

## Data Availability Statement

The raw data supporting the conclusions of this article will be made available by the authors, without undue reservation.

## Author Contributions

JM, JJE, MF, and DV conceived and designed this study. JJE, MF, and JM performed the experiments. DV and JM analysed the data and wrote the manuscript. All authors have read and approved this manuscript.

## Conflict of Interest

The authors declare that the research was conducted in the absence of any commercial or financial relationships that could be construed as a potential conflict of interest.

## Publisher’s Note

All claims expressed in this article are solely those of the authors and do not necessarily represent those of their affiliated organizations, or those of the publisher, the editors and the reviewers. Any product that may be evaluated in this article, or claim that may be made by its manufacturer, is not guaranteed or endorsed by the publisher.

## Supplementary Material

Supplementary Figure 1 Alignment of 24 Sanger sequences of *THCAS* from plants of breeding populations MX-CBD-11 and MX-CBD-707.
